# Focus on Mucosal-Associated Invariant T Cell Antigens Fueling Biliary Liver Injury

**DOI:** 10.1016/j.jcmgh.2026.101846

**Published:** 2026-07-03

**Authors:** Josias J. Mürle, Magdalena Filipowicz Sinnreich

**Affiliations:** Liver Immunology Laboratory, Department of Biomedicine, University and University Hospital of Basel, Basel, Switzerland; Liver Immunology Laboratory, Department of Biomedicine, University and University Hospital of Basel, Basel, Switzerland; Department of Gastroenterology and Hepatology, Basel University Medical Clinic, Cantonal Hospital Basel-Land, Liestal, Switzerland

In this issue of *Cellular and Molecular Gastroenterology and Hepatology*, Nordhus and colleagues^1^ provide fascinating new insights into the role of mucosal-associated invariant T (MAIT) cells and their bacterially derived antigens in biliary inflammation. MAIT cells represent a major semi-invariant immune cell population in the human liver, accounting for up to 40% of liver-resident T lymphocytes.^2^ Restricted by the MHC class I-related molecule MR1, MAIT cells recognize microbial metabolite antigens, most notably the riboflavin biosynthesis intermediate 5-(2-oxopropylideneamino)-6-D-ribitylaminouracil (5-OP-RU),^3^ that places them at the interface of immune responses occurring at the gut–liver axis. A growing body of evidence has highlighted the diverse functions of MAIT cells, encompassing antimicrobial defense, immunoregulation, tissue repair, and profibrogenic activity. These findings suggest that MAIT cells play a multifaceted role in maintaining hepatic homeostasis while also contributing to the pathogenesis of liver disease.^4^

MAIT cells appear to undergo profound alterations in chronic cholestatic liver diseases. In patients with primary sclerosing cholangitis (PSC) and primary biliary cholangitis, MAIT cells are markedly reduced and exhibit an exhausted phenotype in the circulation, while being enriched within portal tracts and preserved within bile ducts.^5^, ^6^, ^7^

However, until recently, the intrahepatic phenotype of MAIT cells in these diseases remained poorly characterized, and the contribution of bacterial-derived MAIT cell ligands to shaping this phenotype was largely unexplored. This knowledge gap is addressed by the authors of the current study,^1^ who investigated MAIT cell responses and associated inflammatory infiltrates in the livers of mice following surgical intrabiliary administration of either the potent MAIT cell agonist 5-OP-RU or fixed *Escherichia coli*. Notably, this work builds on previous findings from the same group demonstrating that bile from patients with PSC contains MAIT cell–stimulatory ligands,^8^ thereby providing a strong rationale for exploring the consequences of local MAIT cell activation within the biliary compartment.

Because MAIT cells are relatively scarce in the murine liver, the authors employed the MAIT-enriched congenic *MR1*^+^ B6-MAIT^CAST^ strain (∼2% intrahepatic MAIT cells) and the transgenic iVα19 Cα^-/-^ Tg strain, the latter harboring up to 25% MAIT cells and more closely reflecting the situation in human liver. *E* *coli* administration induced transient biliary inflammatory infiltrates that were not MAIT-specific and were accompanied by a mild fibrogenic response. In contrast, 5-OP-RU triggered robust intrahepatic MAIT cell activation in iVα19 Cα^-/-^ Tg mice, together with marked inflammatory infiltrates, indicating that high-dose antigen stimulation is sufficient to induce liver inflammation when MAIT cells are abundant. This response was accompanied by early fibrogenic activation, reflected by increased ⍺-smooth muscle actin expression. Although 5-OP-RU also activated MAIT cells in *MR1*^+^ B6-MAIT^CAST^ mice, no activation of other inflammatory cell populations or biliary infiltrates was observed, indicating that MAIT cell abundance is needed to drive biliary inflammation.

The authors further characterized hepatic MAIT cells by single-nucleus RNA sequencing, revealing expansion of liver-resident MAIT cells and a shift toward an MAIT17 phenotype in 5-OP-RU–treated iVα19 Cα^-/-^ Tg mice. This subset, defined by retinoic acid-related orphan receptor gamma t expression and high interleukin17 production,^9^ has been implicated in fibrogenesis. Consistent with this phenotype, 5-OP-RU stimulated activation-associated pathways, including T-cell receptor (TCR) and cytokine signaling, whereas non-MAIT T cells remained largely unresponsive, underscoring the selective effect of 5-OP-RU. In addition, monocyte-derived cells and Kupffer cells emerged as the principal sources of MR1 transcripts in the murine liver. Notably, cholangiocytes also expressed detectable MR1, supporting previous human ex vivo studies demonstrating their capacity to function as antigen-presenting cells for MAIT cells.^10^^,^^11^

By demonstrating that intrabiliary exposure to MAIT cell antigens is sufficient to induce cholangitis, the study provides important mechanistic insight into how MAIT cells may contribute to biliary inflammation. The findings further suggest that the elevated levels of MAIT cell–stimulatory ligands previously identified in PSC bile could represent a pathogenic driver of local immune activation. Collectively, the data support a model in which MAIT cell–dependent cholangitis arises from the interplay between antigen availability, adequate intrahepatic and peribiliary MAIT cell frequencies, and local antigen presentation within the biliary epithelium.

These murine findings need to be interpreted in the context of the human liver. In contrast to mice, where 2 distinct MAIT cell subsets have been described, MAIT cells in the healthy human liver appear to form a continuum of activation states rather than discrete populations.^12^ Nevertheless, combined TCR- and cytokine-mediated stimulation, likely occurring within the inflammatory liver microenvironment, has been shown to induce the emergence of a distinct interleukin-producing MAIT cell population.^12^ Notably, this subset seems enriched in the livers of patients with PSC,^13^ alongside increased MAIT T helper 17–phenotype associated TCR clonotypes in peripheral blood, further supporting a potential role for this population in PSC pathogenesis.

In summary, the current findings identify IL17-skewed MAIT cell activation in response to elevated levels of biliary MAIT cell agonists as a potential mechanism contributing to cholangitis and early fibrogenic responses in the liver. These findings expand the current understanding of biliary immune responses and further underline MAIT cells as important participants in the immunopathogenesis of chronic cholangiopathies.
